# Geographic differences in body size distributions underlie food web connectance of tropical forest mammals

**DOI:** 10.1038/s41598-024-57500-5

**Published:** 2024-03-23

**Authors:** Lydia Beaudrot, Miguel A. Acevedo, Daniel Gorczynski, Nyeema C. Harris

**Affiliations:** 1 Program in Ecology and Evolutionary Biology, Department of BioSciences, Rice University, Houston, TX USA; 2 Department of Wildlife Ecology and Conservation, University of Florida, Gainesville, FL USA; 3 Applied Wildlife Ecology Lab, School of the Environment, Yale University, New Haven, CT USA; 4 Department of Integrative Biology, Michigan State University, East Lansing, MI USA

**Keywords:** Ecology, Zoology, Ecology, Environmental sciences

## Abstract

Understanding variation in food web structure over large spatial scales is an emerging research agenda in food web ecology. The density of predator–prey links in a food web (i.e., connectance) is a key measure of network complexity that describes the mean proportional dietary breadth of species within a food web. Connectance is a critical component of food web robustness to species loss: food webs with lower connectance have been shown to be more susceptible to secondary extinctions. Identifying geographic variation in food web connectance and its drivers may provide insight into community robustness to species loss. We investigated the food web connectance of ground-dwelling tropical forest mammal communities in multiple biogeographic regions to test for differences among regions in food web connectance and to test three potential drivers: primary productivity, contemporary anthropogenic pressure, and variation in mammal body mass distributions reflective of historical extinctions. Mammal communities from fifteen protected forests throughout the Neo-, Afro-, and Asian tropics were identified from systematic camera trap arrays. Predator–prey interaction data were collected from published literature, and we calculated connectance for each community as the number of observed predator–prey links relative to the number of possible predator–prey links. We used generalized linear models to test for differences among regions and to identify the site level characteristics that best predicted connectance. We found that mammal food web connectance varied significantly among continents and that body size range was the only significant predictor. More possible predator–prey links were observed in communities with smaller ranges in body size and therefore sites with smaller body size ranges had higher mean proportional dietary breadth. Specifically, mammal communities in the Neotropics and in Madagascar had significantly higher connectance than mammal communities in Africa. This geographic variation in contemporary mammalian food web structure may be the product of historical extinctions in the Late Quaternary, which led to greater losses of large-bodied species in the Neotropics and Madagascar thus contributing to higher average proportional dietary breadth among the remaining smaller bodied species in these regions.

## Introduction

Food webs, which describe multi-level trophic interactions between consumers and resources, play critical roles in the maintenance of diversity^1,2^. They can be represented as ecological networks where species are nodes that are connected through their interactions as edges^3^. Decades of research have uncovered some generalities based on individual food web networks. For example, a subset of species in a community is often highly interactive and therefore well connected in a food web^3^. As such, trophic cascades, trait-based cascades, and eco-evolutionary feedbacks can propagate across multiple species in a community through direct and indirect effects on population dynamics^4^. Most of our understanding of food web networks, however, is derived from single-site studies or microcosm experiments, which inhibits generalizations across scales and hampers broader predictions of how global change will impact trophic networks and ecosystem functioning^5^.

Understanding variation in food web structure over large spatial scales, its driving factors, and its eco-evolutionary consequences is an emerging research agenda in food web ecology^6^. Even though food webs are a critical component of ecological communities, research on community assembly has largely concentrated on horizontally defined communities consisting of a single trophic level, such as plants^7–9^. Multitrophic studies are needed to identify general patterns that can advance community ecology towards a comprehensive understanding of community assembly^10^. Food web studies require information on trophic interactions, which are lacking for many taxa. Mammals, however, have more published studies describing predator–prey interactions than other taxa^4^ making mammals an excellent taxonomic group for testing for drivers of food web structure over large spatial scales. Tropical forests occur in equatorial regions around the world and therefore provide the opportunity to compare mammal food web structure in communities within the same biome on different continents. Most tropical forest mammal species occur exclusively on one continent and therefore similarity in community structure among biogeographic regions is not due to shared species.

Unique ecological and evolutionary histories within biogeographic regions have been shown to relate to the taxonomic, phylogenic, and functional structure of tropical mammal communities^11–13^. However, multiple components of tropical mammal community structure are consistent among biogeographic regions^14–16^, which suggests the potential for food web network structure to be similar among regions as well. For example, a global comparison of mammal communities found that tropical forests around the world contain functionally similar mammal communities despite differences in biogeographic history^14^. At least two studies have demonstrated consistent proportions in the relative species richness of coarse dietary guilds for tropical mammal communities in different regions^15,17^, which suggests that broadly similar habitats have led to similar trophic composition. In addition, Mendoza and Araujo^16^ identified six trophic structures for mammal communities globally that clustered together based on trophic guilds. Trophic structure categories mapped spatially to humid tropical, seasonal tropical, semiarid, temperate, and boreal areas, which suggests that similar trophic structures occur under similar environmental conditions in distant biogeographic regions^16^, yet the extent to which tropical mammal predator–prey interaction network structure is similar among regions remains unknown.

A large body of research has explored the relationship between food web network complexity and stability (for review, see^18^), and the density of species interactions or connectance has been used to quantify network complexity for over 50 years^19–22^. Connectance is quantified as the proportion of observed links in a network relative to the possible number of links and it is referred to more generally as network density^23^. Given that connectance describes the links between each species in a food web and the species it feeds on, it can be interpreted as the mean proportional diet breadth of all species in a food web^24^. Connectance can be mechanistically modelled as an emergent consequence of individual foraging behavior with optimal foraging constraints on diet breadth. Specifically, connectance of the animal portion of food webs can be reproduced from a model that assumes the most profitable prey species is always consumed, and that predator diet breath is the number of prey (in order of profitability) that maximize the rate of energy intake^25^. There is some evidence that connectance is largely constant among food webs within a habitat type^26–28^, further suggesting the potential for similarity in food web connectance among tropical forest regions.

Connectance is a critical component of food web robustness to species loss. For example, a secondary extinction occurs when the removal of a species from a food web (e.g., due to simulated extinction) causes a remaining consumer species to go extinct due to loss of its resources^26^. The removal of highly connected species causes higher rates of secondary extinctions and faster fragmentation of food webs than the random loss of species^3,29,30^. Food webs with lower connectance have been shown to be more susceptible to secondary extinctions^3,31^ Greater susceptibility to extinctions occurs when species are less densely linked to each other because fewer species have to be lost before consumers lose all of their resources. Furthermore, a common measure of structural robustness is the proportion of species that have to be removed from a food web to lose more than half of the species in the web (R_50_)^32^ and food web structural robustness increases logarithmically as food web connectance increases^33^. Therefore, identifying geographic variation in food web connectance and its ecological and anthropogenic drivers may provide insight into community robustness to species loss.

Here our objectives are to 1) test for differences in tropical mammal food web connectance among biogeographic regions, and 2) test three potential drivers of variation in contemporary tropical mammal food web connectance: primary productivity, contemporary human pressure, and variation in mammal body mass distributions reflective of historical extinctions. Primary productivity—the rate at which energy is converted into biomass, typically through photosynthesis—plays an essential role across scales of biological organization and understanding its influences on trophic interactions has been a long-standing focus in ecology^34–36^. Primary productivity has been shown to explain the proportion of basal species within food webs^37^ and food chain length in natural and experimental systems^38–40^. Nevertheless, how food web connectance varies in response to gradients in primary productivity is unknown. If higher productivity contributes to greater specialization (i.e., via the niche diversity mechanism^41^), then we predict that mammal communities in more productive environments will have fewer observed food web links relative to possible links and therefore have lower food web connectance.

As the biosphere changes more rapidly now than any time in human history, changes in land use can alter fundamental relationships between consumers and their resources thereby altering food web dynamics^42^. For example, human activities have been associated with simplified mammal trophic structures in Europe and eastern North America^16^. Protected areas are a cornerstone of conservation and arguably provide the best opportunity to compare food web structure among locations while minimizing human impacts^43^, yet protected areas can vary greatly in the degree of contemporary anthropogenic threats that can mediate food web structure, including illegal wildlife poaching and isolation due to land cover change^44^. Losing species from protected areas may reduce the range of body sizes if the smallest or largest species are extirpated. If the body size range shrinks with species loss, then fewer physical (i.e., sized-based) feeding constraints are expected to restrict predation causing the remaining species in the food web to have more densely linked predator–prey interactions^45^. Therefore, species loss from a community is predicted to result in increased connectance^46^. If contemporary anthropogenic pressure influences food web structure within tropical forest protected areas, then we predict that sites with more hunting, greater habitat fragmentation, and higher surrounding human density will have higher connectance.

Historical losses of large-bodied mammals may also influence contemporary food web structure given that size-selected extinction has been a long-term trend^47^. Importantly, the severity of large mammal extinctions has varied among continents. African large mammal communities remain the most intact among tropical regions because these communities survived the last glacial maximum relatively unscathed^48,49^. Similarly, southern Asia has been less affected by historical extinctions than other regions^50^. In contrast, South America lost more than three-fourths of megafaunal genera during the late Quaternary^50^ and Madagascar lost most megafauna in the last 3000 years^51^. If historical extinctions of large-bodied mammals have influenced modern food web structure, then we predict that sites with smaller average body sizes and smaller body-size ranges will have higher connectance.

Here we show that food web connectance is similar in tropical African and Asian mammal communities but significantly higher in Neotropical and Malagasy communities, and differences in species’ body size ranges significantly predicted food web connectance. More possible predator–prey links relative to possible links were observed in communities with smaller body size ranges, which indicates larger mean proportional diet breadth of species in a food web when body size ranges were smaller. As the range of body sizes increased, predators on average interacted with a smaller proportion of prey species. To our knowledge this is the first study to connect differences in tropical forest mammal body sizes to food web structure. Furthermore, these results suggest that differences among regions in tropical mammal food web connectance may stem from regional differences in the historical extinction of large-bodied mammals, which were more severe in the Neotropics and in Madagascar than in the Afro- and Asian tropics.

## Methods

### Study taxa and sites

To identify mammal community composition, we leveraged observational data from tropical protected areas around the world. Specifically, we used species occurrence lists generated from camera trap images collected by the Tropical Ecology Assessment and Monitoring Network (TEAM). TEAM has used large-scale arrays of cameras to systematically monitor terrestrial (i.e., ground-dwelling) mammals. All TEAM study sites have followed a single camera trapping protocol in which 60 camera traps were deployed in gridded arrays for 30 days a year for multiple years^52^. TEAM has monitored species with average body mass greater than 100 g that spend a large proportion of time on or near the ground because these are the species that could be identified and monitored using terrestrial camera traps.

**Figure 1:**
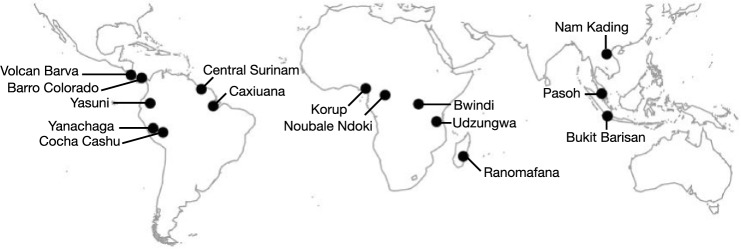
Location of TEAM study sites. Information about each site is available in Table S1.

We used published occurrence lists of the species TEAM monitored from 15 protected areas in the Neo- (N = 7), Afro- (N = 4), Asian (N = 3) and Malagasy (N = 1) tropics (Fig. 1, Table S1). All study sites were located within 21.5 degrees from the equator, contained tropical evergreen forest, and had mean annual precipitation of at least 1350 mm. These sites included a total of 393 mammal populations from 183 unique species representing 115 genera from 42 families in 16 orders (for complete list, see^53^). Mammal richness ranged from 21 to 35 species except for the Malagasy site, which had 13 species. Most genera (94.5%) were found in only one region with 5 genera occurring in two regions (*Atherurus* spp*.*, *Herpestes* spp*.*, *Hystrix* spp*.*, *Potomochoerus* spp*.*, and *Tapirus* spp*.*). *Panthera* spp. was the single genus that occurred in three regions. Only two species occurred in more than one region: the leopard (*Panthera pardus*) in Africa and Asia, and the bushpig (*Potamochoerus larvatus)* in Africa and in Madagascar, where it was introduced in pre-colonial times^54^. Thus, each tropical region had species pools that were essentially unique.

### Trophic networks

Fundamentally, food webs are networks of consumer-resource interactions among a group of organisms, and they can be quantified using network theory where a species is a node, and an interaction is an edge^55^. Predator–prey interactions are inherently directed because the direction of the interaction is from the predator to the prey. In predator–prey directed networks, species cannot interact with themselves, which therefore assumes that cannibalism, which is rare in mammals^56^, does not occur, but intraguild predation is allowed.

To generate predator–prey interaction networks for each TEAM site, we constructed an overarching mammal food web for each site from predator–prey interactions documented in published literature. Specifically, we used a meta-web approach^57^, which is a common approach in food web studies over large spatial scales^58,59^. Site-specific food webs were subsampled from the meta-web using site-specific mammal community composition data. The meta-web approach reduces differences in interaction sampling bias among sites that could affect comparisons otherwise^55^. We recognize that communities defined by camera trap observations lack small or arboreal mammals and other taxa. Nevertheless, almost all multitrophic studies rely on a subset of interactions among organisms^10^.

Quantifying trophic interactions exhaustively is a common challenge in food web ecology. Generally, we have a partial understanding of food web networks because even intense sampling can result in incomplete detection of interactions^60^. Trophic interactions can be difficult to detect because they are infrequent, they occur between rare species, or both^61^. Existing models have demonstrated that information on species traits, such as body size, can provide a fairly accurate description of empirical food web networks^62,63^. We therefore constructed a second meta-web that included all predator–prey interactions identified from the published literature as well as possible interactions based on either 1) another species in the same genus documented in a predator–prey interaction with the predator or 2) species that fell within the size distribution of prey known to be consumed by the predator. We conducted all analyses using both food web definitions and results were qualitatively the same. All results using the second more inclusive meta-web are presented in the supplementary material. Site-specific food web networks from each of the two meta-webs are shown in Figure S1.

We quantified food web structure using directed connectance because predator–prey interactions are inherently directional (i.e., predators eat prey, but prey do not eat predators). This network metric is defined as the proportion of observed interactions relative to the possible number of interactions. It varies between 0, which represents a lack of predator–prey interactions and 1, which represents all possible predator–prey interactions within a network. We calculated directed connectance using the CollectionPCS function from the R package “cheddar”^64^.

### Generalized linear modeling

To test the extent to which mammal communities in tropical forests worldwide vary in their food web structure*,* we conducted a generalized linear regression that modeled directed connectance of the mammal food web at each TEAM study site as a function of the categorical predictor variable biogeographic region (i.e., the Neotropics, Afro-tropics, SE Asian tropics, or Madagascar). We then conducted a second generalized linear model to test the relative importance of predicted drivers of food web structure. The second model used food web connectance as the response variable and we tested TEAM study site level predictors of connectance. The seven predictor variables were species richness, primary productivity, the percent of populations hunted, forest edge density as a measure of forest fragmentation, human density, body size range, and median body size as described below. All predictor variables were measured for the time-period that corresponded with camera trap monitoring.

We included mammal species richness as a predictor variable to account for its potential influences on food web structure given its positive relationship with connectance^65^. We used published values of the normalized difference vegetation index (NDVI) for TEAM sites^17^ as a proxy for plant productivity and predictor of food web connectance. To test for contemporary human impacts on food web structure, we used published data for each TEAM site on the mammal species hunted^53^, which we summarized as the percent of mammal species hunted per site, forest edge density^53^, which is a measure of forest fragmentation, and human density^17^. All three of these measures of contemporary human impacts varied among sites (Figure S3). For the previously published hunting data, field managers at each TEAM site were surveyed about hunting within the protected area where the core TEAM camera trap sampling occurred. Specifically, species lists were distributed to managers who marked whether each species on their site list was hunted, not hunted, or whether they did not know^53^. Because processes beyond protected area limits may affect wildlife inside the parks, we accounted for anthropogenic pressure on the broader landscapes for each TEAM site using the concept of the zone of interaction (ZOI). The ZOI is the spatial extent with the potential to strongly influence biodiversity based on systematic quantification of surrounding watersheds, migration corridors and human settlements^66^. To evaluate forest fragmentation for the ZOI at each TEAM site, we used previously published values for the density of forest edges in the ZOI^53^. These values were based on a 75% threshold for the 2000 forest cover layer in the Global Forest Change product^67^ and considered the gain and loss layers in the product to calculate a forest-non-forest map for each site for 2012. Data were then filtered to set a minimum patch size of 990 m^2^ and the proportion of forested landscape^68^) was extracted from the forest cover data for each TEAM ZOI using the “ClassStat” function in the SDMTools library in R^69^. Lastly, we used published human density values for the ZOI^17^ as a measure of anthropogenic pressure. The distribution of hunted species, habitat fragmentation, and human population density for the TEAM sites is shown in Figure S2. Finally, to examine the influence of body size distributions on food web structure, we used published species-level body size data^53^ to calculate the range in body sizes and the median body size for the mammal species observed at each TEAM study site.

For both generalized linear regression models, we used a quasibinomial distribution with a logit link function, which is a common approach to model proportional responses^70^. We fit both models using a maximum likelihood approach. The maximum correlation among predictor variables was 0.61 (Figure S2). Continuous predictor variables were scaled and centered in the regression model to allow direct comparison of standardized coefficient estimates and aid convergence. Differences were considered statistically significant for 95% confidence intervals of odds ratios that did not include one with positive effects greater than one and negative effects less than one^71^. To assess model fit, we compared observed and predicted values from the fitted models^72^. We first conducted both regressions using the food webs comprised only of predator–prey interactions known from published literature. We then conducted the two regressions described above using the meta web with known interactions as well as possible interactions. All analyses were conducted in R^73^.

## Results

**Figure 2:**
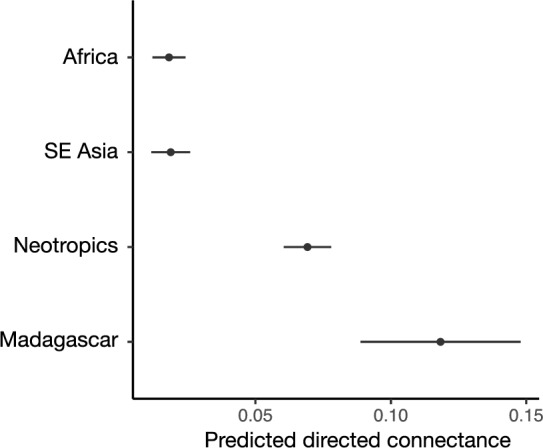
Directed connectance predicted for each geographic region from the generalized linear model testing for differences among regions. The plot displays the mean estimates and their 95% confidence intervals. Food webs used only predator–prey interactions known from the literature. Results were qualitatively similar for food webs that used known interactions as well as possible interactions (Figure S4).

Our first goal was to test for variation in mammal food web connectance in tropical forest biogeographic regions and we found significant differences based on 95% confidence intervals of odds ratios that did not contain one. Directed connectance was significantly higher in Neotropical (odds ratio = 4.04, 95% CI [2.79, 5.84]) and Malagasy mammal communities (odds ratio = 7.29, 95% CI [4.67, 11.37]) than in African mammal communities (Fig. 2), which have been the least impacted by megafaunal extinctions^48^. Directed connectance in Asian mammal communities, however, did not differ significantly from African mammal communities (odds ratio = 1.04, 95% CI [0.62, 1.74]). Model results were qualitatively similar for directed connectance calculated from the second meta-web, which included published predator–prey interactions as well as possible interactions (Figure S4).

**Figure 3:**
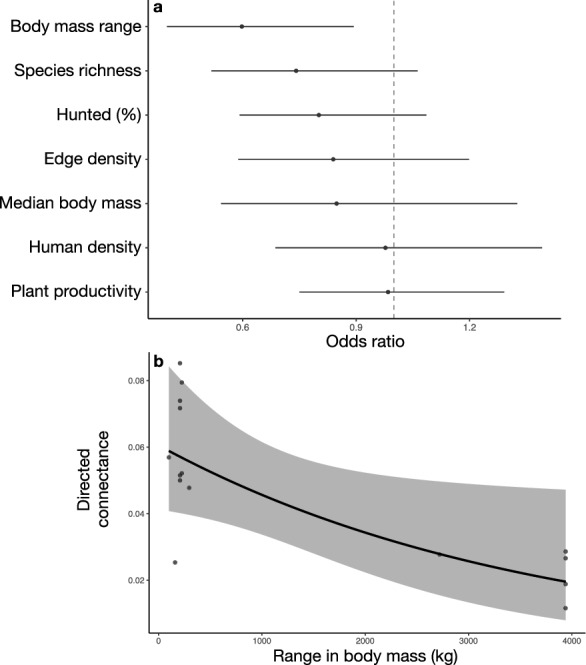
Model results from the generalized linear regression testing for predictors of food web connectance. (**a**) The coefficient plot displays the mean odds ratio estimates and 95% confidence intervals for the standardized predictor variables. We consider an effect statistically significant when the 95% confidence interval does not include one, which is shown by the vertical dotted line. Food web connectance was significantly predicted by range in body mass. (**b**) Model predictions of directed connectance (solid line) with 95% confidence intervals (gray shading) for the observed range in body mass values. Points show the partial residuals of the observed body mass range data accounting for the effects of the other model predictors. Results were qualitatively similar for food webs that used known interactions as well as possible interactions (Figure S5).

**Figure 4:**
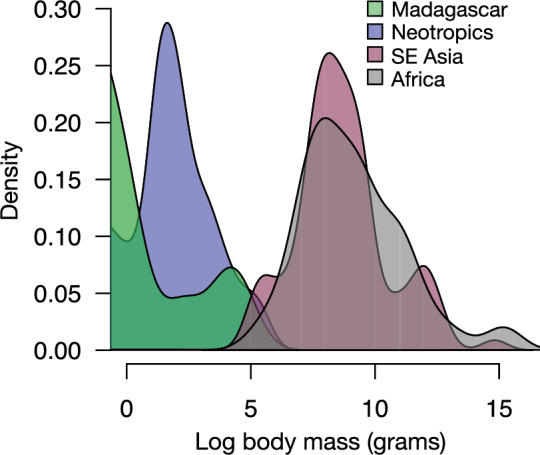
Distributions among geographic regions for the body mass of the 393 mammal populations in this study, which included 110 African populations, 89 Asian populations, 13 Malagasy populations, and 181 Neotropical populations. Note that the term population is used rather than species because some species occurred at multiple study sites.

Our second goal was to identify predictors of food web connectance in tropical forest mammal communities. We found that only body size range significantly predicted food web connectance (Figs. 3 and 4). Larger mammal community body size ranges were significantly associated with lower directed connectance (odds ratio = 0.69, 95% CI [0.54, 0.89]) but larger median body sizes were not significantly associated (odds ratio = 0.93, 95% CI [0.67, 1.28]).

In contrast with body size range, none of the anthropogenic pressure variables significantly predicted food web connectance. Specifically, we did not find a significant effect for the percent of mammal species hunted (odds ratio = 0.87, 95% CI [0.70, 1.08], forest edge density (odds ratio = 0.93, 95% CI [0.73, 1.19]), or human density (odds ratio = 1.02, 95% CI [0.81, 1.27]). We also did not find a significant effect for NDVI (odds ratio = 0.98, 95% CI [0.81, 1.18]) or species richness (odds ratio = 0.87, 95% CI [0.68, 1.12]). Model results were qualitatively similar for directed connectance calculated from the second meta-web that included published and possible interactions (Figure S5).

## Discussion

The two main goals of this study were to test for differences in tropical forest mammal food web connectance among biogeographic realms, and to identify potential drivers of food web connectance. The mammal communities investigated in this study have consistent trophic guild species richness among sites^17^. Nevertheless, we found that their food web structure differed significantly among continents. Thus, observed generalities among large mammal trophic structures^16^ do not necessarily extend to mammal food web networks. Furthermore, connectance was not constant throughout the tropical forest biome despite evidence that connectance is constant within some habitat types^26^. Even though the functional diversity of the mammal communities in this study was significantly higher in protected areas with more primary productivity^74^, we did not find a significant relationship between primary productivity and mammal food web connectance. Despite variation in contemporary anthropogenic pressure within the protected areas, we did not find evidence that hunting, forest fragmentation, or human density contributed significantly to variation in food web connectance. Instead, food web structure was significantly and most strongly predicted by the range in body mass within mammal communities. Communities with smaller body-size ranges occurred in regions that have faced more historical extinctions and a larger proportion of the possible predator–prey interactions in their food webs occurred.

Extinctions during the late Quaternary primarily affected large-bodied terrestrial mammals^50^. The Neotropics and Madagascar have been most heavily affected by megafaunal extinctions among tropical regions and consequently have smaller-bodied species today^50,51^. In Madagascar, for example, the subfossil record includes at least 17 species of now extinct giant lemurs that ranged in body size from 10 to 200 kg^75^. Notably, we found significantly higher food web connectance in the Neotropics and Madagascar than in Africa. At the same time, the distribution of body sizes in Asian and African populations in this study largely overlapped while food web connectance in Asian and African mammal communities did not differ significantly. Riede et al.^45^ described how species loss should lead to communities with higher connectance because remaining species would be more densely linked with each other in their trophic interactions. In support of this prediction, we found that tropical forest mammal communities with more historical extinctions of large bodied species had higher food web connectance, which indicates that more of the possible interactions among species were observed. We suggest that differences in body sizes among regions that have been exacerbated by historical extinctions have likely contributed to contemporary differences in tropical mammal food web structure worldwide.

Other differences in historical biogeography among regions, such as species compositions from differing evolutionary lineages, may have contributed to the significant differences in food web connectance. For example, the island of Madagascar has been relatively isolated from mainland Africa for over seventy million years and the unique evolutionary history of mammals resulted in high levels of endemism^76^. Madagascar has historically had lower mammal species richness and smaller bodied species than continental tropical forests^47,75^. Therefore, historical differences in body size that existed prior to megafaunal loss may have influenced food web connectance. Additionally, given that we constructed food webs from published data, it is possible that differences among regions in the availability of predator–prey interaction data contributed to the results. Still, our results were consistent whether food webs were defined using known predator–prey interaction links or possible interactions based on taxonomy and body size, which suggests that the observed patterns were not likely due to a regional sampling artifact.

The lack of evidence for contemporary human pressure on food web structure within tropical protected areas potentially suggests that protected areas provide benefits for biodiversity by preserving the trophic relationships within food webs that are key for maintaining ecosystem functioning^1^. Nevertheless, the absence of evidence of contemporary human pressure on food web connectance is not evidence of absence. There are now multiple lines of evidence documenting anthropogenic impacts on the protected tropical forest mammal communities analyzed in this study. For example, despite the fact that functional redundancy in tropical forest mammal communities can buffer declines in functional diversity when species loss is random^77^, recent extirpations within the study areas have resulted in reduced mammal functional diversity due to the loss of species with unique functional traits, including large-bodied carnivores and specialist insectivores^74^. Moreover, human pressure affects the underlying dynamics that determine mammal occurrences within the protected areas: survival probability near protected area boundaries is significantly lower when human density is high^78^. High human density also influences spatial associations among mammal species^79^, which in turn impact local colonization and extinction dynamics^80^, with the potential to impact species interactions and ecosystem function.

The mounting evidence demonstrating anthropogenic impacts on protected tropical forest mammal functional diversity, occupancy dynamics, and spatial associations raises the question of why significant effects on food web connectance were not found in this study. One possible explanation is that the large differences in body size ranges among regions and the resulting differences in proportional dietary breadth surpassed any impacts of current anthropogenic pressure on food web connectance. Limitations of the measures used for food web connectance and contemporary human pressure may also have been a contributing factor. For example, food web connectance was determined by the presence or absence of links between species as is most often the case^26^. Due to data availability, it did not incorporate quantitative data, such as interaction strengths, which might respond more sensitively to human pressure. In addition, hunting was quantified as a summary statistic of the percent of mammal populations hunted at each protected area due to data availability, yet the impacts of hunting on mammal abundances vary among species and locations^81^. Nevertheless, the lack of evidence for contemporary human pressure on food web connectance is not necessarily inconsistent with the human-induced loss of functional diversity at some TEAM sites. Connectance declines significantly with the diversity of species interactions within food webs because there is more dissimilarity in species interactions when food webs are sparsely connected^82^. Interaction diversity, however, is not significantly correlated with conventional functional diversity indices, such as functional richness, evenness, dispersion or Rao’s Q^82^, which suggests that connectance is likely not strongly correlated with conventional functional diversity indices either. The relationship between food web structure and functional diversity has only recently begun to be investigated formally and more work integrating trait-based approaches with food web networks is needed^83^.

Despite a lack of evidence that contemporary anthropogenic pressure significantly affected tropical mammal food web structure within protected areas, historical human impacts on large-bodied mammals may have been an important driver of food web differences among regions because increases in human population density best predict mammal extinctions from the Late Quaternary^84^. Furthermore, extinction induced declines in mammal food web complexity over the last 130,000 years became more severe as humans colonized the world^63^. Our finding that tropical forest mammal communities with smaller body size ranges have higher connectance may be due in part to regional variation in human induced extinctions. Additional work is needed to assess whether mammal food web connectance differed among geographic regions prior to human arrival.

Food webs with lower connectance have been shown to be more susceptible to secondary extinctions because food webs are sensitive to the removal of highly connected species^3,31^. Given their lower connectance, African and Asian mammal communities may be more vulnerable to secondary extinctions following the loss of highly connected species despite having maintained more megafauna to date. In the coming decades, African mammal food webs will likely be more intensely affected by land-use change than other regions because projected human population growth rates for sub-Saharan Africa are among the highest in the world^85^. Mammals can strongly influence their environment by changing plant communities, habitat structure, trophic dynamics, and nutrient flows^86^. Indeed, the distribution of animal body sizes within communities affects the ratios of nutrients distributed to plants through animal feces due to set stoichiometric ratios that vary with herbivore body size^87^. Therefore, shifts in the body size distribution of mammals within a community can affect the redistribution of nutrients throughout the landscape. Additional work is needed to test for continental variation in the vulnerability of tropical mammal food webs to extinctions and the resulting consequences for ecosystem functions.

### Supplementary Information

Supplementary Information 1.

Supplementary Information 2.

## Data Availability

All data generated or analyzed during this study are included in this published article and its supplementary information files.
