# Supplementary material for: Geographic differences in body size distributions underlie food web connectance of tropical forest mammals
Source: Sci Rep. 2024 Mar 23;14:6965. doi: 10.1038/s41598-024-57500-5 (PMC10960815; doi:10.1038/s41598-024-57500-5)

Supplementary Information

Title: Geographic differences in body size distributions underlie food web connectance in tropical forest mammals

Authors: Beaudrot, Acevedo, Gorczynski & Harris

Table S1. TEAM study site locations, mammal species richness, and food web directed connectance. See Figure 1 for map of geographic locations. Site codes correspond with mammal food web networks shown in Figure S1.

Region	Code	Site	Country	Mammal Species	Directed Connectance (published only)	Directed Connectance (published or possible)
Africa	BIF	Bwindi Impenetrable National Park	Uganda	26	0.010	0.059
Africa	KRP	Korup National Park	Cameroon	23	0.025	0.074
Africa	NNN	Nouabalé Ndoki National Park	Republic of Congo	35	0.020	0.044
Africa	UDZ	Udzungwa Mountains National Park	Tanzania	26	0.018	0.080
Asia	BBS	Bukit Barisan Seletan National Park	Indonesia	31	0.025	0.071
Asia	NAK	Nam Kading National Protected Area	Laos	24	0.009	0.063
Asia	PSH	Pasoh Forest Reserve	Malaysia	34	0.022	0.089
Madagascar	RNF	Ranomafana National Park	Madagascar	13	0.118	0.160
Neotropics	BCI	Barro Colorado Nature Monument - Soberania National Park	Panama	24	0.061	0.117
Neotropics	CAX	Caxiuanã National Forest	Brazil	23	0.078	0.147
Neotropics	COU	Cocha Cashu - Manu National Park	Peru	31	0.057	0.147
Neotropics	CSN	Central Suriname Nature Reserve	Suriname	29	0.064	0.139
Neotropics	VB	Volcán Barva, Braulio Carrillo National Park	Costa Rica	21	0.087	0.182
Neotropics	YAN	Yanachaga Chimillén National Park	Peru	24	0.071	0.156
Neotropics	YAS	Yasuni National Park	Ecuador	29	0.067	0.150

Figure S1. Mammal food web networks for each TEAM site defined by **a**) predator-prey interaction data collected from published literature and **b**) all predator-prey interactions identified from the published literature as well as possible interactions (see methods). Abbreviations follow TEAM site codes listed in Table S1.

a.

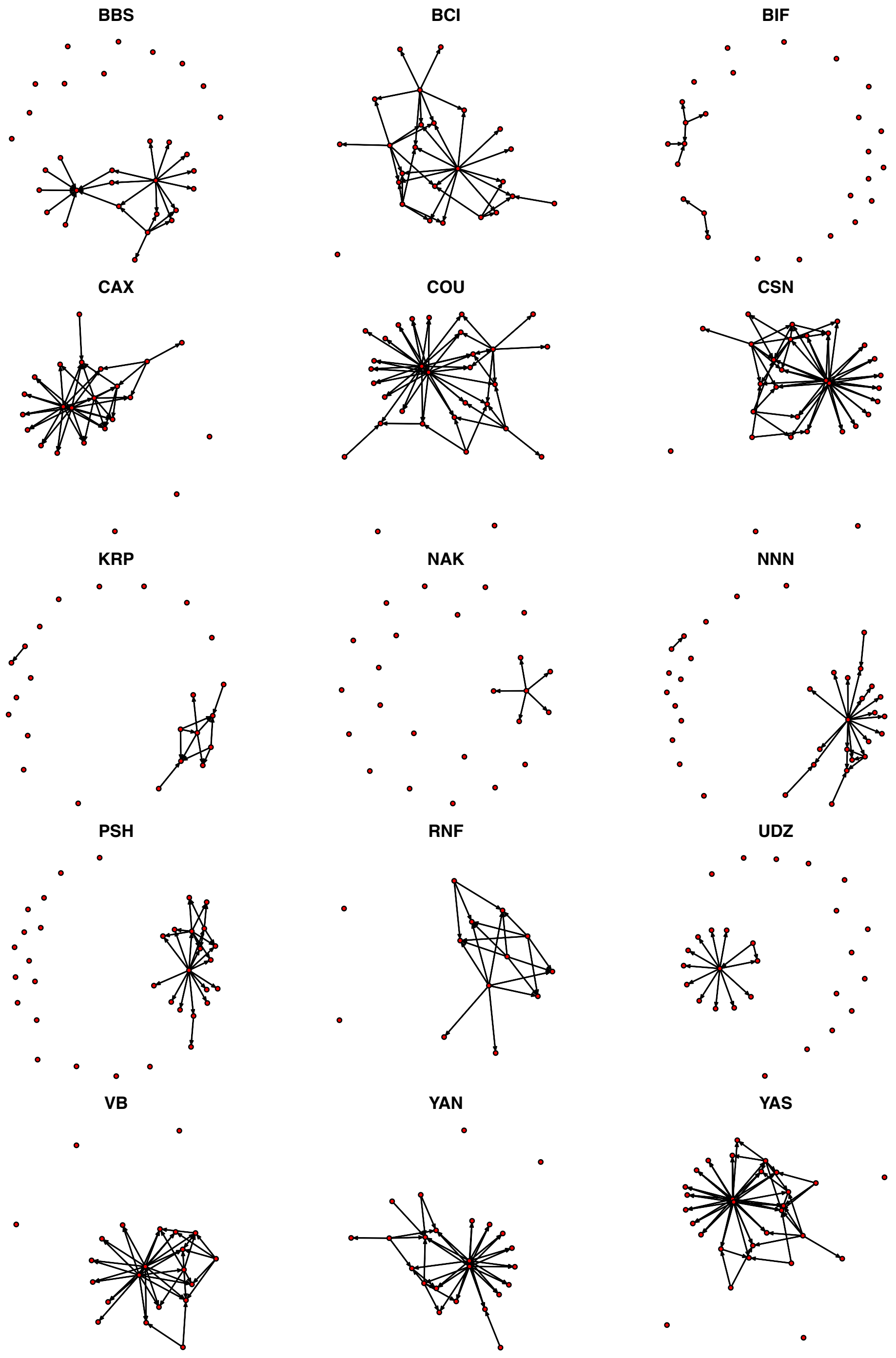

b.

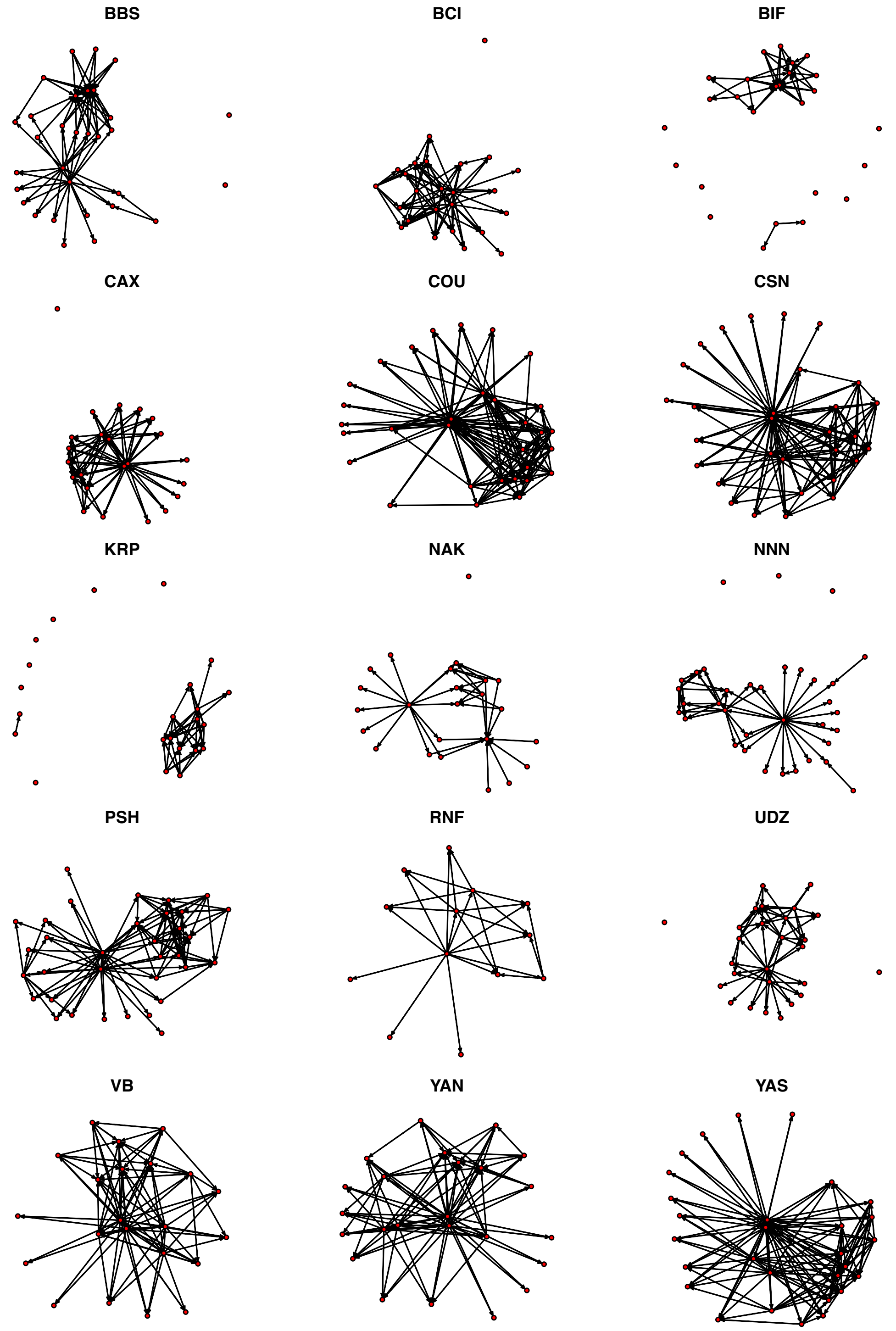

Figure S2. Variation among TEAM sites in the level of anthropogenic disturbance: a) the percent of mammal populations hunted within the protected area, b) habitat fragmentation in the zone of influence as measured by the density of forest edges, and c) human density in the zone of influence. Anthropogenic disturbance data from Beaudrot et al. 2016 *Plos Biology*.

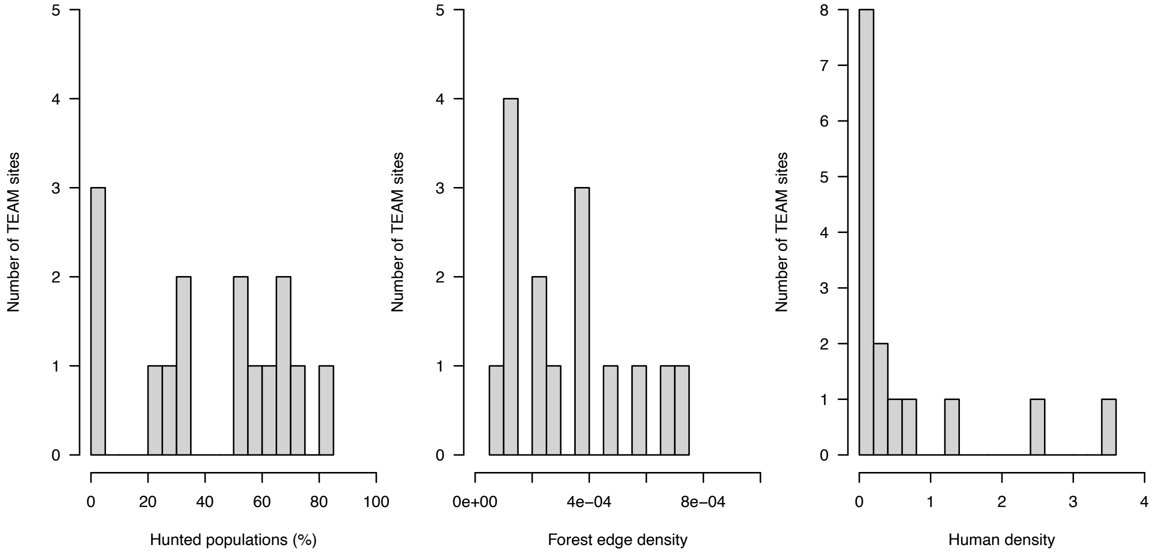

Figure S3. Pairwise relationships among predictor variables. The lower panel displays plots of each pair of predictors. The upper panel displays the correlation for each pair with text size proportional to the correlations. The TEAM study site-level predictor variables were species richness (Richness), the percent of populations hunted (Hunted), human population density in the ZOI (PopDen), range in body size (Range_Body), median body size (Median_BodyS), edge density in the ZOI (EdgeDensity), and primary productivity as measured by NDVI. Correlations among all predictor variables were less than or equal to 0.61.

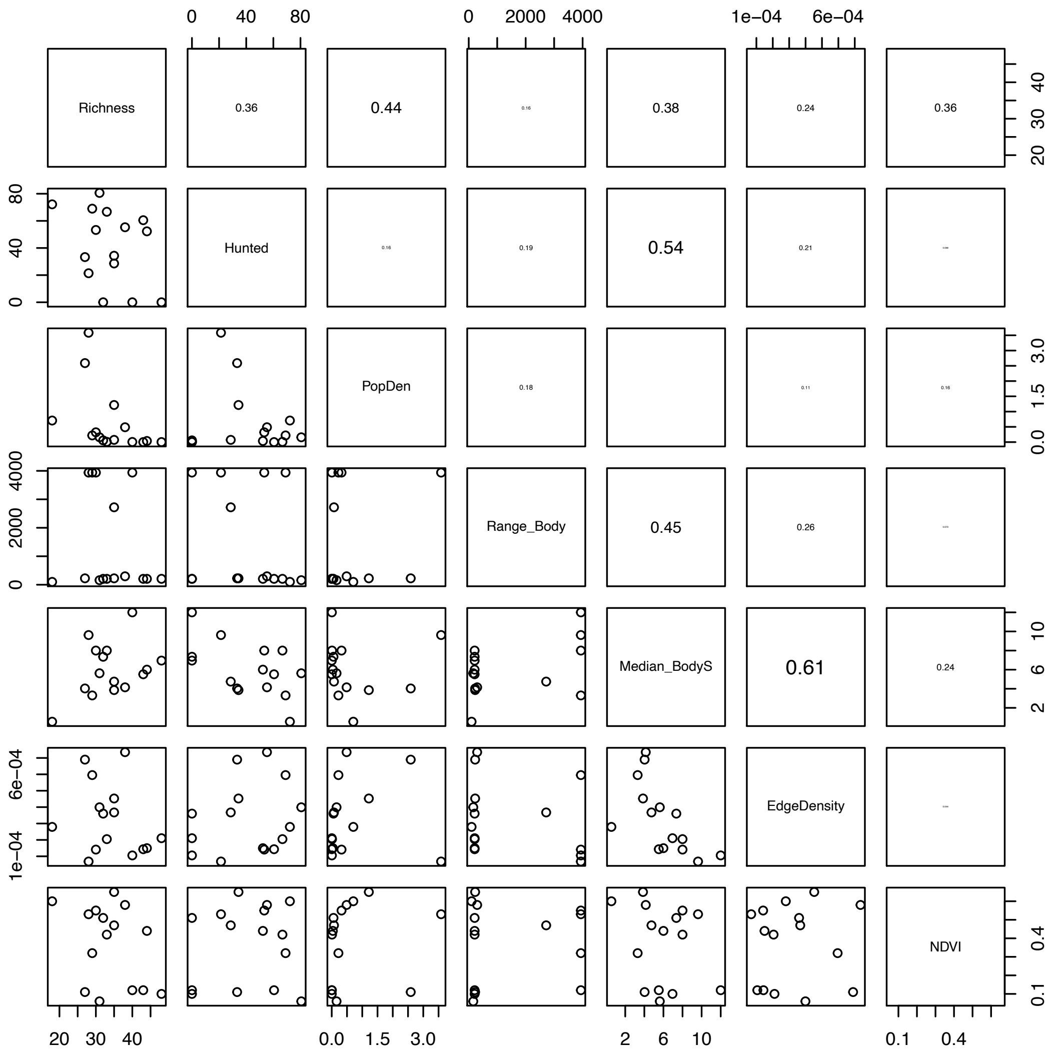

Figure S4. The predicted directed connectance values for each geographic region from the generalized linear regression model using known and possible predator-prey interactions were qualitatively similar to those presented in the main text that only used interactions known from the scientific literature (Figure 2). The plot displays the mean estimates for each region shown by the points and the 95% confidence intervals shown by the thin black lines.

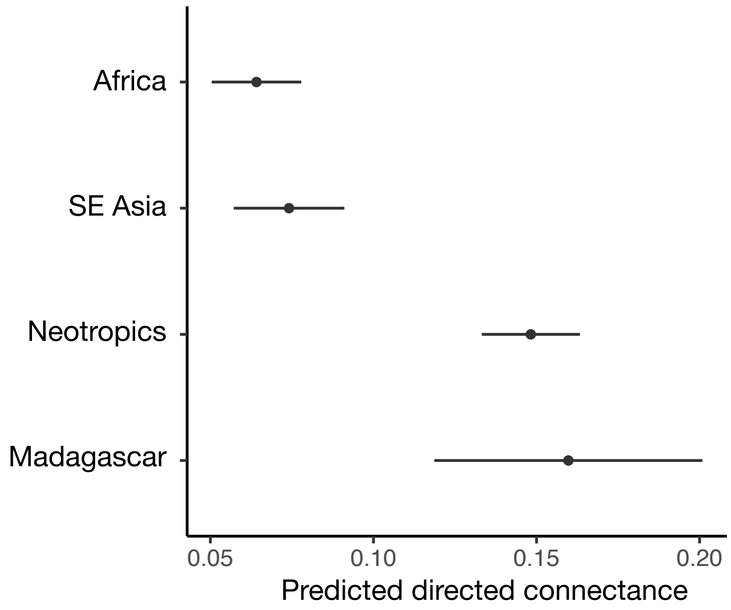

Figure S5. Model results from the generalized linear regression testing for predictors of food web connectance. The model results for directed connectance using known and possible predator-prey interactions were qualitatively similar to those presented in the main text that only used interactions published in the scientific literature (Figure 3). **a**) The coefficient plot displays the odds ratios for the predictor variables shown by the points and the 95% confidence intervals shown by the thin black lines. We consider an effect statistically significant when the 95% confidence interval does not include one, which is shown with the vertical dotted line. Food web connectance was significantly predicted by range in body mass. **b**) Model predictions of directed connectance (solid line) with 95% confidence intervals (gray shading) for the observed range in body mass values. Points show the partial residuals of the observed body mass range data accounting for the effects of the other model predictors.

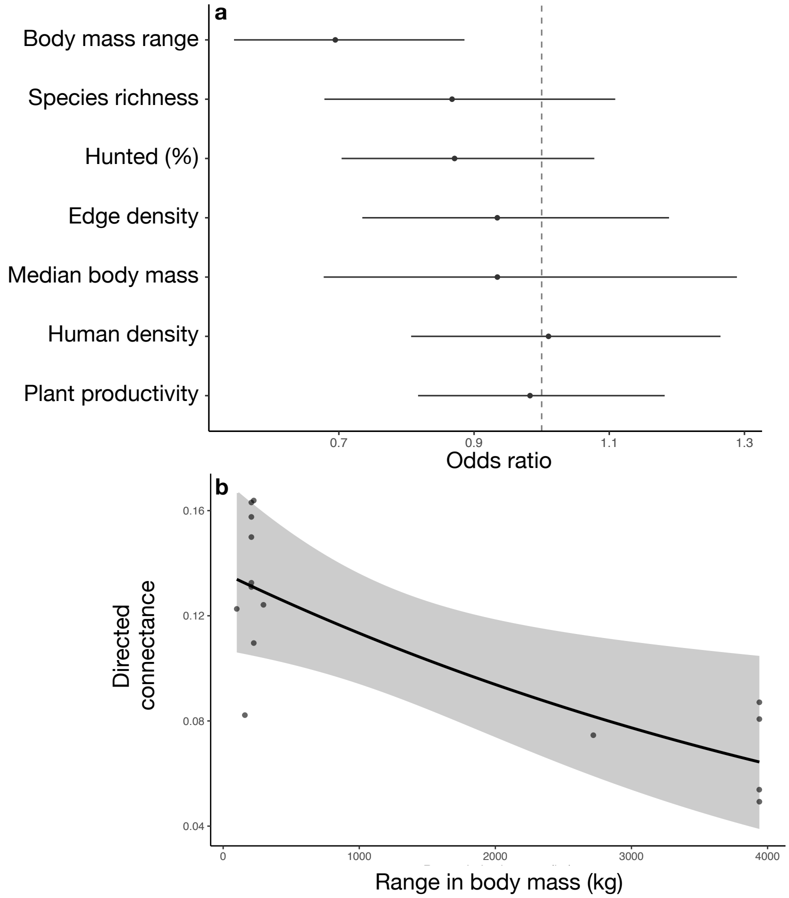

Supplement: Supplementary file 2 — Supplementary Information 2. [file 41598_2024_57500_MOESM2_ESM.pdf]
